# GTX.Digest.VCF: an online NGS data interpretation system based on intelligent gene ranking and large-scale text mining

**DOI:** 10.1186/s12920-019-0637-x

**Published:** 2019-12-20

**Authors:** Yanhuang Jiang, Chengkun Wu, Yanghui Zhang, Shaowei Zhang, Shuojun Yu, Peng Lei, Qin Lu, Yanwei Xi, Hua Wang, Zhuo Song

**Affiliations:** 1 Genetalks Biotech. Co., Ltd., Changsha, 410000 China; 2 State Key Laboratory of High-Performance Computing, College of Computer, National University of Defense Technology, Changsha, 410073 China; 3 NHC key laboratory of birth defects research, prevention and treatment (Hunan Provincial Maternal and Child Health Care Hospital), NO.53 Xiangchun Road, Changsha, 410008 Hunan China; 4 Cytogenetics and Human Molecular Genetics Laboratories, Royal University Hospital, Saskatoon, SK Canada; 5 Hunan Provincial Maternal and Child Health Care Hospital, Changsha, 410073 China

**Keywords:** NGS data interpretation, Gene prioritization, Neural network, Text mining, Distributed parallel computing

## Abstract

**Background:**

An important task in the interpretation of sequencing data is to highlight pathogenic genes (or detrimental variants) in the field of Mendelian diseases. It is still challenging despite the recent rapid development of genomics and bioinformatics. A typical interpretation workflow includes annotation, filtration, manual inspection and literature review. Those steps are time-consuming and error-prone in the absence of systematic support. Therefore, we developed GTX.Digest.VCF, an online DNA sequencing interpretation system, which prioritizes genes and variants for novel disease-gene relation discovery and integrates text mining results to provide literature evidence for the discovery. Its phenotype-driven ranking and biological data mining approach significantly speed up the whole interpretation process.

**Results:**

The GTX.Digest.VCF system is freely available as a web portal at http://vcf.gtxlab.com for academic research. Evaluation on the DDD project dataset demonstrates an accuracy of 77% (235 out of 305 cases) for top-50 genes and an accuracy of 41.6% (127 out of 305 cases) for top-5 genes.

**Conclusions:**

GTX.Digest.VCF provides an intelligent web portal for genomics data interpretation via the integration of bioinformatics tools, distributed parallel computing, biomedical text mining. It can facilitate the application of genomic analytics in clinical research and practices.

## Background

To interpret a VCF file and determine the disease-cause gene, traditional interpretation tools are required at first. Recently, some gene prioritization and literature evidence search tools are also provided to save manually search time.

### Traditional interpretation

Emerging genome and exome sequencing technologies and platforms are producing massive amounts of sequencing data globally. The software pipeline for a typical workflow from raw sequencing data (FASTQ format) to a variant call format (VCF) file is relatively mature [1]. For instance, BWA + GATK is frequently recommended and employed [2]. However, it remains a challenging task to interpret sequencing data in terms of highlighting the disease-causing gene from thousands of mutated genes in one’s VCF file. The traditional interpretation is: firstly, VCF file using software such as ANNOVAR [3], snpEff [4], VEP [5], and VAT [6]; secondly, keeping the variants in coding region and splicing site and removing synonymous variants; thirdly, choosing the variants with high pathogenicity scored by predicting software, such as SIFT [7], PolyPhen2 [8], CADD [9], DANN [10] etc., and with low allele frequencies in population genomes from 1000 genome project [11], U10K project [12], ExAC Project [13], NHLBI GO Exome Sequencing Project [14], etc. Some filtration operations are required on the above annotation results to reduce the number of candidate gene mutations.

### Disease gene prioritization

After an initial step of filtration, there are usually a large number of mutated genes remain (from hundreds to thousands) left for manual curation. To accelerate this tedious process, several phenotype-based gene ranking tools were developed. Accepting patients’ standardized phenotype descriptive terms (HPO, Human Phenotype Ontology) [15] as input, these software rank mutated genes according to their pathogenicity. Thus, users can review genes following this prioritization. There are many advanced gene ranking software according to literature. Such Exomiser [16], AMELIE [17], Phenomizer [18], Phevor [19] and others [20–23].

Most of previous gene ranking methods use gene-phenotype relationships curated from OMIM [24] and OrphaNet [25] to prioritize a list of candidate genes according to the given clinical phenotypes. Phenomizer [18] and Phevor [19] are the typical methods of this kind. These methods require continuous comprehensive manual curation to improve the gene-phenotype database.

Exomiser [16] comprises a phenotype-driven prioritization procedure for disease-gene discovery in the field of Mendelian diseases. Genes in a VCF file are ranked according to clinical relevance assessed via one of three phenotype-driven algorithms (PHIVE, PhenIX or hiPHIVE) or by a random-walk algorithm that evaluates the vicinity of the genes to members of disease-gene family according to the protein-protein interactome. Users can integrate Exomiser with other interpretation functions easily.

AMELIE [17] against the above methods by using data mining techniques to ranks the candidate genes. It parses hundreds of thousands of abstract and full-text articles to find an underlying diagnosis to explain a patient’s phenotypes given the patient’s candidate gene list. AMELIE uses published paper to get the gene-phenotype relationship automatically, no manually generated database is required. Yet, it does not considerate the pathogenicity of mutations.

### Literature evidence inspection

Even the software can prioritize candidate genes, only about 50% of real pathogenic genes can be ranked in TOP 20 in practical application. Users still need a lot of time to decide which one is the real pathogenic gene or mutation. Generally, a manual review relies heavily on a comprehensive inspection of relevant literature, which involves a lot of search and reading work. In a typical setting, it might involve dozens of articles in order to obtain information about a single SNP on multiple diseases, which makes the processing time consuming and tedious.

To reduce the time of search and reading academic papers, some search engines, such as LitVar [26] and other methods [27, 28], are implemented using text mining techniques to extract the relationship between gene (variant) and disease from tens of millions of literature deposited in PubMed Central (PMC).

Thus, to interpret a VCF file, the user needs to run several tools in order to obtain disease-causing genes and literature evidence. Some tools are hard to use, some of them can’t provide expected results. So, it is still a laborious work for gene interpretation.

### Implementation

In this paper, we developed a system that provides an intelligent portal for genomics data analyses. We named it GTX.Digest.VCF. It is an online NGS data interpretation system based on intelligent gene prioritization and large-scale text mining. For gene prioritization, a multilayer neural network is adopted to analyze the pathogenicity of genes and mutations. The neural network gives a disease-causing score (GTX.score) for each mutation to rank the genes and mutations in a VCF file. For large-scale text mining, a parallel text mining approach is applied on all MEDLINE abstracts and PMC open-access full-texts in advance to find the relationships between genes/mutations and diseases, the mined results are stored as a database in GTX.Digest.VCF for fast queries.

GTX.Digest.VCF provides both prioritized candidate genes (mutations) and mined literature evidence. The system website takes variant calling data (in VCF format generated via GATK) and phenotypes as input. Users can use the system to set filtration conditions related to genotypes and phenotypes. GTX.Digest.VCF then ranks genes and variants using a trained neural network model. The system output is a ranked list of genes and mutations with text mining annotations, presented on an interactive web page. For each given gene/mutation, GTX.Digest.VCF also shows the related mined results in the form of pie charts and evidence sentences. The results of the system can also be exported as a formatted report.

The GTX.Digest.VCF system is publicly available as a free web portal. The system is hosted on AWS and it utilizes several parallel computing methods to ensure the scalability of the system. To note, NGS data analyses are computational intensive. There is a server node which provides web services to show the interpreting results, while all data analyses process are running in a distributed way on computing nodes. For each interpreting task, we applied a new AWS EC2 with 8 CPU cores as a computing node to process the data analyses in a parallel way automatically. Depending on the size of the input VCF file, it normally takes half an hour for the data analysis before you can view the results.

One can use GTX.Digest.VCF without pre-registration. For users who have the requirement for data privacy and data management, registering a free private account is highly recommended. The GTX.Digest.VCF server has been up since 2017-12-30. During system tests, we have analyzed over 100 cases. About 20 clinical doctors and analysts have evaluated the system.

With the help of GTX.Digest.VCF, bioinformaticians and clinical doctors can easily analyze variant data of a patient, interpret the results with literature evidence and produce a test report that can be understood by the patient. In this way, the time costs of genomics tests and results interpretation can be greatly reduced and the reports will be more reliable. We will continuously improve the system and aim to maintain the system for at least ten years.

### System design and architecture

**Fig. 1:**
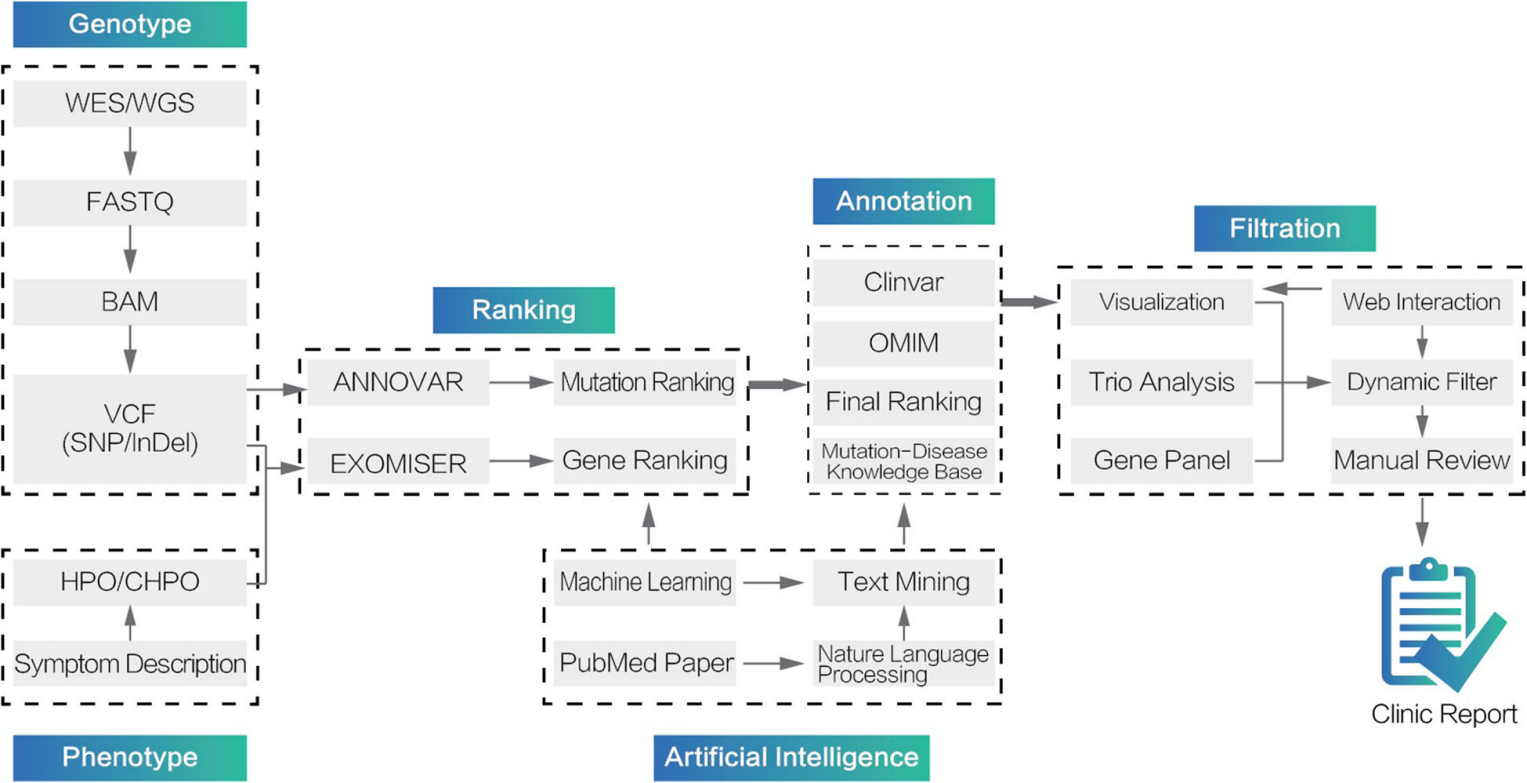
The architecture of GTX.Digest.VCF system

The software architecture of GTX.Digest.VCF is illustrated in Fig. 1. The system support two analytic types: single VCF files analysis for a singleton case and three GVCF files analysis for a trio (family) case.

After the input of VCF files and HPO terms, GTX.Digest.VCF first runs ANNOVAR to get the traditional interpretation results. Then some of the traditional interpretation results are arranged as the mutation features, and input to the trained neural network to calculate GTX.score for the mutation. GTX.score is used to generate the final gene and mutation ranking results. In annotation step, the database of Clinvar [29], OMIM [24] (more database including HGMD and PharmGKB will be added in the future), and mutation-disease knowledge base generated by text mining are integrated together for gene annotation. Eventually, the visualized results are shown on the result web page, where gene panel and dynamic filtering functions can be operated through web interaction. Manual marks for specific mutations are supported to generate the final report.

In GTX.Digest.VCF, running ANNOVAR and retrieving text mining database requires a large amount of computational resources. To improve the scalability, GTX.Digest.VCF runs these parts in a distributed parallel way. To analyze each VCF file, GTX.Digest.VCF will apply an AWS EC2 instance automatically to execute these parts. After finishing the tasks, the results will be returned to the server node for further process, and the EC2 instance will be terminated automatically.

The EC2 instance of the server node is r4.xlarge configured with 4 computing cores, 30.5GB memory and 80GB hard disk. The computing node instance is r5d.2xlarge configured with 8 computing cores, 64GB memory and 300GB SSD. Ubuntu operating system is installed on both kinds of nodes.

### System usage

**Fig. 2:**
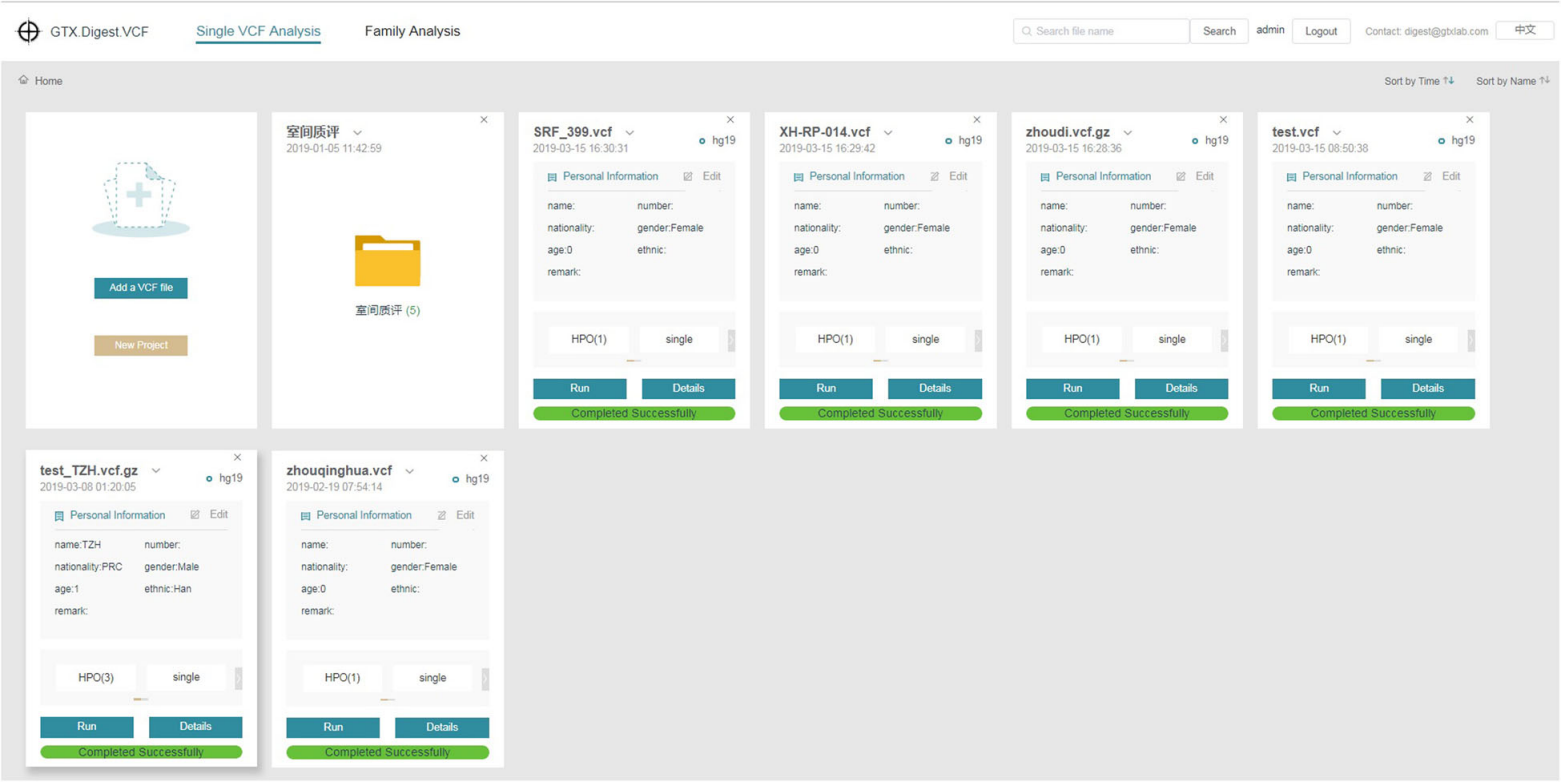
The managing web page of VCF cards

GTX.Digest.VCF requires genotype inputs in VCF file format and phenotype inputs in the form of HPO terms. After uploading a VCF file, a VCF card is generated (Fig. 2 shows the managing web page of VCF cards). The reference genome (hg19/hg38) will be captured automatically from the VCF file (if failed, manual input is required) and displayed on the right corner of the card. Personal information filled in by users is shown in the middle of the card. The “Gender” must be filled in by users. Other personal information like name, age, nationality, ethnic, accession number, and the remark is optional.

Next, four buttons including “HPO”, “single/family”, “splicing site” and “Group frequency” provide parameter settings for running NGS interpretation. Clicking “Run” button will start the analyzing procedure, a progress bar at the bottom of the card shows the running progress. Clicking the “Details” button, the system will turn to the result page of the current VCF file.

The following parameters are essential for data analyses. (i) “HPO”: This option is used for inputting phenotypes corresponding to the VCF file. Users can input the name or the accession number of the phenotype. Predictive typing is supported when inputting the phenotype information. (ii) “Group frequency”: This option provides a selection from a set the genome of populations according to the patient’s background. By default, the most popular three genomes of populations in practical applications are selected. (iii) “Splicing site”: This parameter is the number of intronic sites away from an exon/intron boundary. The threshold is set to 10 by default. Besides, a |z-score| suggesting how strongly splicing site variants affect RNA splicing is offered and the default value is 1.8 in the system. GTX.Digest.VCF keeps the splicing site variants located between the exon/intron boundary and “Splicing site” value with |z-score| large than the set value.

Based on the above parameters, GTX.Digest.VCF will run the analyzing procedure by clicking the “Run” button. It takes about 30 min on average. The larger the size of the VCF file, the longer the running time required.

**Fig. 3:**
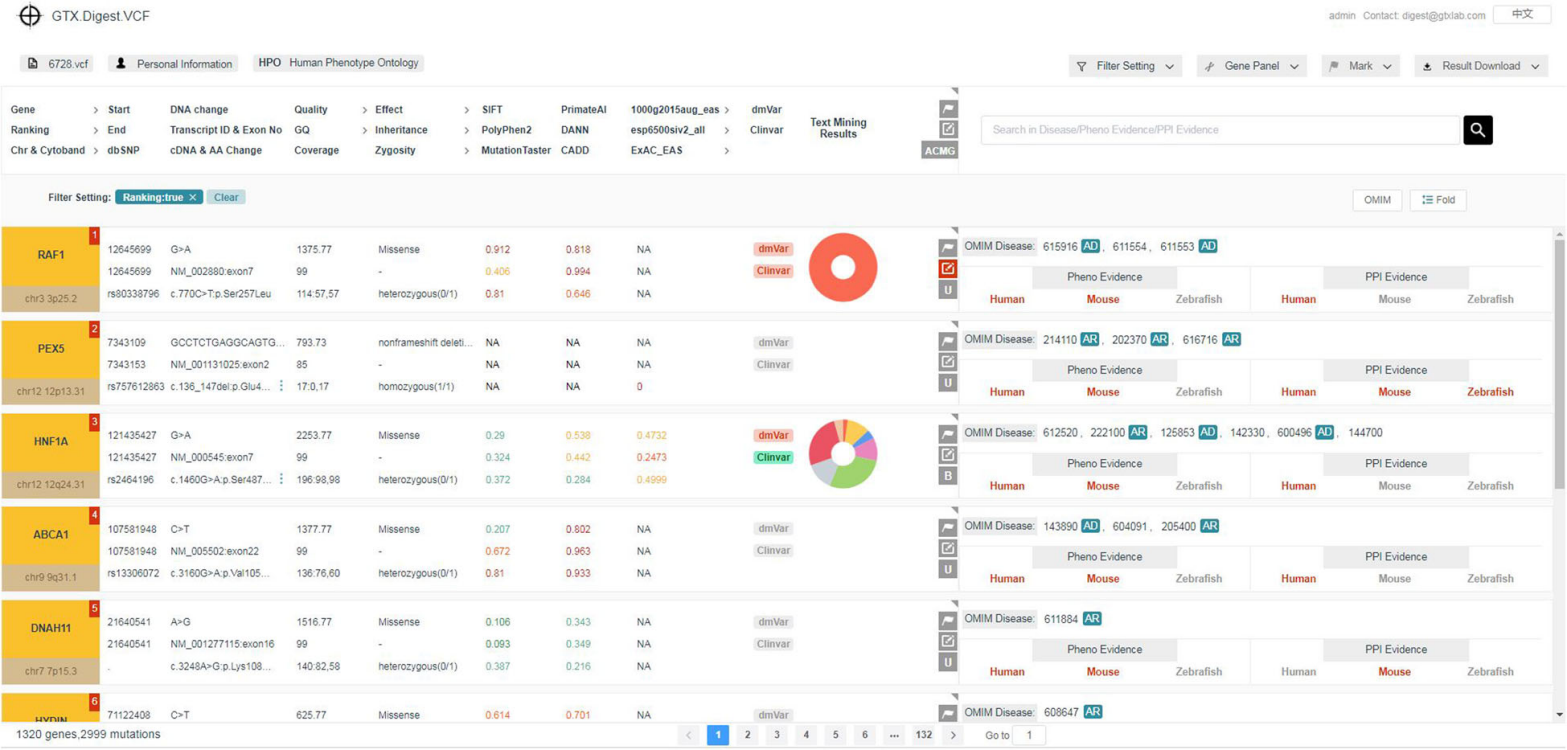
The result web page

All analyzing results are shown on the result web page (see Fig. 3) in a ranked way according to the pathogenicity of genes. The interpretation results of each mutation are list in one line. Only the most pathogenic mutation is shown in folding mode. For the gene has more than one mutation, the results of other mutations can be seen in unfolding mode by double-clicking the current result line, or by clicking the “unfold” button. If the “ranking” option on the left-top of the page is set to “no”, the results will be ranked by research popularity according to the text mining results. The resulting line of each mutation has 12 columns (three elements per column) of information, 6 of which can be used for dynamic filtering. The top of the result page gives the meaning of the corresponding results in each column. Users can download the analyzing results in CSV format through “Result Download”.

### Ranking genes and mutations using multilayer neural network

Exomiser and AMELIE are phenotype-driven prioritization procedure for disease-gene discovery in the field of Mendelian diseases. Exomiser combines the results of three phenotype-driven algorithms and the protein-protein interactome and generates a combined score for the pathogenicity of individual genes. While some important results, such as pathogenicity in Clinvar database, data mining results, and the conservation of DNA sequence [30, 31], are not considered in Exomiser. AMELIE use data mining techniques to ranks the candidate genes. Yet it does not considerate the pathogenicity of mutations.

To improve the prioritization of gene and mutation, a multilayer neural network approach is provided in our system. For each mutation, features that are fed to the neural network include: phenotypes in the gene-related diseases, phenotypes in the protein-protein interactome, conservation score of the mutation generated via the GERP++ tool [32], pathogenicity results in Clinvar database, the maximum MAF result from different population genomes, results of predicting software, data mining results obtained from PubMed abstracts, etc. The output results of the neural network are the pathogenicity score of the mutation called GTX.score. The value of GTX.score is in [0, 1]. The higher the score value, the more deleterious the related mutation.

The EGAD00001001355 dataset of the Deciphering Developmental Disorders (DDD) project provides several hundreds of VCF files with related phenotypes and real pathogenic genes. Two popular gene ranking algorithms, AMELIE and Exomiser, are applied on 305 DDD VCF files where the pathogenic mutation are SNP variants. For each VCF file, the given pathogenic mutation is a training example, and its GTX.score value is set to 1. We choose other 40 genes with the closest rank values prioritized by AMELIE and Exomiser from each VCF file. If the selected gene has only one mutation, this mutation is a training example. For the gene with multiple mutations, the most deleterious mutation is chosen as a training example. The GTX.score of each training example is set as follows:

$$ \mathrm{GTX}.\mathrm{score}={\gamma}^{x-1} $$

where *x* is the average gene rank value of AMELIE and Exomiser for the mutation *m* in the corresponding VCF files.

$$ x=\frac{\mathrm{Rank}\left(\mathrm{AMELIE},m\right)+\mathrm{Rank}\left(\mathrm{Exomiser},m\right)+\mathrm{Rank}\left(\mathrm{Phenomizer},m\right)}{3} $$

*γ* is the decay factor, the default value is set to 0.99.

The trained model is integrated into GTX.Digest.VCF system. During the interpretation process, the features of each mutation are inputted into the trained neural networks and a GTX.score valued in [0, 1] is obtained. The GTX.score of each gene is the highest GTX.score of all mutations located on this gene. All gene are ranked according to their GTX.score at last as the prioritization results.

### Large-scale text mining on PubMed literature

#### Accurate information retrieval

The text mining task in GTX.Digest.VCF can be divided into two steps, named entity recognition (NER) of diseases and mutations as well as relation extraction (RE) between them. Thus, we need to locate descriptive information about diseases, mutations and their correlations from unstructured texts.

NER aims at identifying text strings that refer to specific biomedical concepts (entities). It is a classical problem in the field of biomedical text mining. Diseases and mutations are the entities of interest in our study. We employed DNorm [33] and tmVar (2.0) [34], which are two state-of-the-art tools for disease NER and mutation NER, respectively. DNorm outperforms other tools with a macro-averaged F-measure of 0.809. In addition, DNorm not only specifies strings as disease mentions but also normalize those mentions to the MEDIC vocabulary for diseases (which combines MeSH and OMIM data). tmVar 2.0 can extract and normalize variant mentions to RSIDs (unique identifiers for variants used in dbSNP) with a high F-measure of ~ 90%. The source code of the two tools is both freely available online.

Extracting semantic relationships from biomedical literature is a challenging task. Rule-based methods are still proved to be an effective way. PKDE4J [35] is an outstanding representative with an 81% F-measure for relation extraction. PKDE4J provides both NER and relation extraction function. However, PKDE4J does not support entity normalization. Therefore, we feed NER results from DNorm and tmVar into PKDE4J for relation extraction. In addition, we found some bugs and fixed them. The original code did not reset the container, which was designed to collect the features that match the predefined rules, before dealing the next instance. This inevitably results in keeping redundant information and leads to invalid relation extraction. Therefore, we implemented a function to clear the features in the container after finishing processing each instance. Besides, we redefined the input format as well as the output format. According to the new input format, the char offsets of the targeted entities in a sentence was fed to PKDE4J except for the sentence text. In this way, we preferred to avoid calling the embedded entity extracting module which may bring unexpected error to the subsequent processing. Moreover, the improved PKDE4J offered the shortest dependency path and the trigger verb word, besides the relationship between the two candidate entities.

By integrating NER and RE, our text mining approach can locate evidence sentences that involve both diseases and variants and highlight the key relation word (if available) in those sentences.

#### Efficient data processing

There are over 800,000 MEDLINE abstracts and nearly 400,000 free PMC full-texts according to a PubMed query ((“mutation”[MeSH Terms] OR “mutation”[All Fields]) AND “loattrfree full text”[sb]). Therefore, the computation time required for above-mentioned NER and RE processing is enormous. In our tests, it took about 1 min on average to process each full-text article on a commodity server. That means over 400,000*1/60 = 6667 h (or 278 days) processing time in total, which is unacceptable.

Additionally, the computation complexity of text mining is greatly affected by the length and content of each article. Therefore, the processing time of each article varies a lot, which highlights the importance of a carefully designed load-balancing strategy.

To mine the tremendous biomedical literature efficiently, and balance the load among the computing nodes, we employed a distributed parallel algorithm, DTM (Distributed Text Mining) to tackle this computational challenge on cloud platforms. In DTM, one server node and tens or hundreds of computing nodes work together for the large-scale text mining task.

A server daemon resides on the server node which is responsible for text mining task distribution. Several computing processes reside on each computing node, and all computing processes work independently for NER and RE computing. Different computing node supports a different number of processes according to its hardware configuration. In our task, each computing process requires at least one CPU and 10GB memory. Each computing process fetches a task (paper ID) from the server daemon, then mines the entities and relations in the paper.

The server daemon maintains a task queue, which is initialized with all paper IDs to be processed. On receiving a task request from a computing process, the daemon will assign the first paper ID in the task queue to the requested process, and remove the paper ID from the queue. If the task queue is empty, the daemon will send a NO-TASK answer.

When a computing process becomes idle, it will request a paper ID from the server daemon for computation. Then the process will do text mining for the paper, including named entity recognition for mutations, disease, and extracting the relations between mutations and diseases. If a computing process gets a NO-TASK answer from the server daemon, that is, there is no paper waited to be processed, the computing process will exit automatically. A computing node will be released by itself if all its computing processes exit normally. New computing nodes (homogeneous or heterogeneous computing nodes) can join the text mining work whenever the task queue on the server is not empty. While a computing node meets an error and quits its mining job unexpectedly, the server will detect the error and reload the unfinished papers into the task queue.

**Fig. 4:**
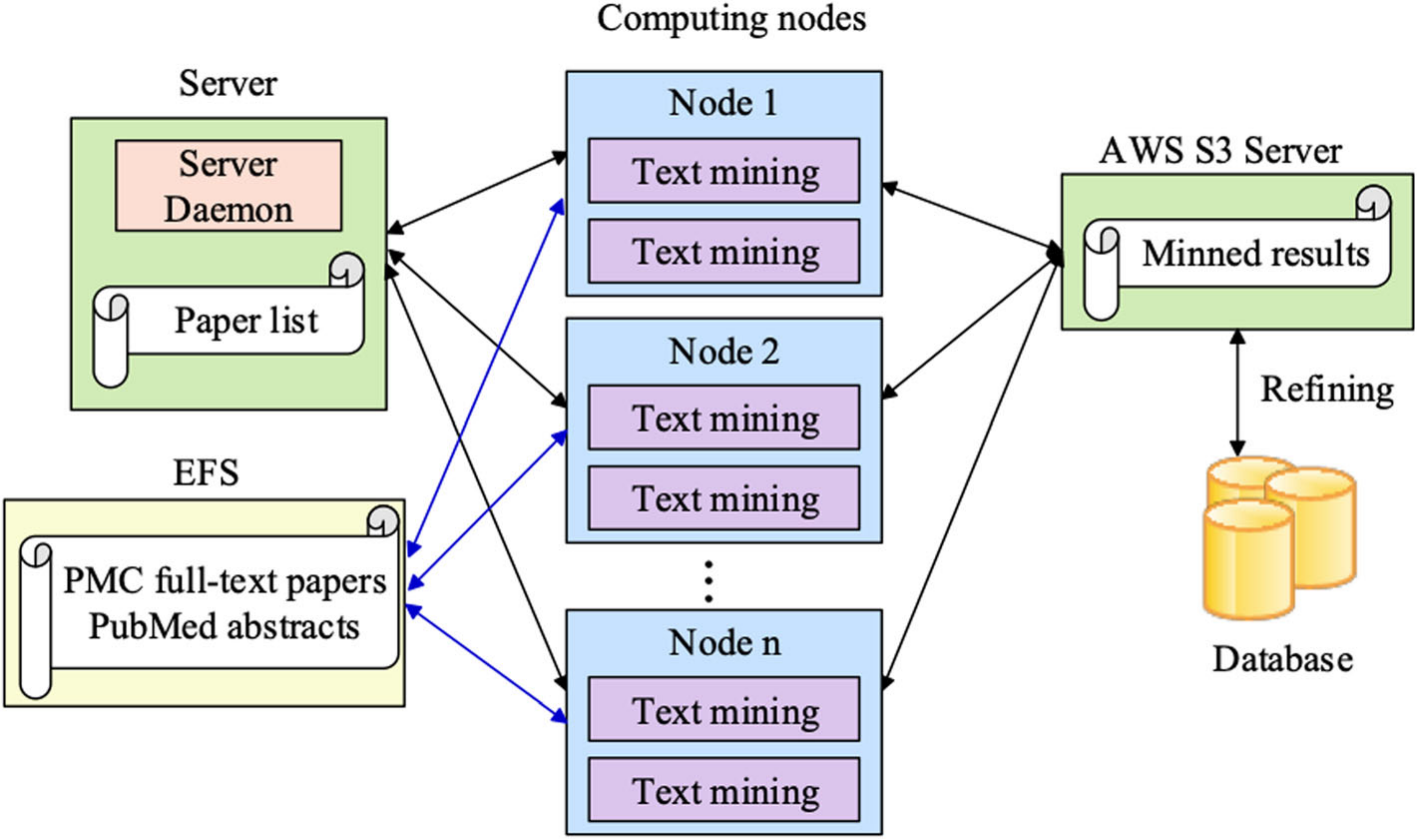
DTM algorithm running on the AWS platform

Figure 4 illustrates our DTM algorithm running on the AWS (Amazon Web Services) cloud platform. We applied an EC2 m5.large machine as the server node and 60 EC2 r4.xlarge machines as computing nodes. The server daemon on the server node distributes tasks. Two computing processes were running on each computing node. All literature data, including PMC full-text and MEDLINE abstracts, were saved on Amazon EFS (Elastic File System) storage and shared by all computing nodes. Once a computing process finished its current text mining task, it uploaded the mined results of the task to AWS S3 Server before requesting a new task.

After the text mining process, post-processing is applied to the mined results to generate a mutation-disease knowledge base. This knowledge base is used to provide literature evidence for the relationship between mutations and diseases in the GTX.Digest.VCF system. Given a variant (either using the RSID or a pre-specified format), all the related diseases in the mined database are shown through a two-level pie chart and highlighted sentences in GTX.Digest.VCF system.

### Enriched filtering functions

GTX.Digest.VCF provides enriched filtering functions to help users focus on mutations of interest easily and quickly.

#### Filter settings

Users can set their own filtering conditions to decrease the number of mutations shown on the result pages: (1) Chromosome: This option is useful when users want to see the variants on some specific chromosomes, where ChrM option means mitochondrion; (2) Variant type: 11 variant types can be detected by the system. The other variants not belonging to these 11 types will be set to “unknown” type. Through selecting the interested variant type, the variants of the other types will be filtered out. (3) Zygosity: Zygosity is the characterization of an individual’s hereditary traits in terms of gene pairing in the zygote from which it developed. There are three zygosity options, heterozygous, compound heterozygous and homozygous. Setting the option if you know the zygosity of the pathogenic variants. (4) Inheritance: This filtering function is useful for Trio (or family) analysis to choose inheritance modeling of the variants. For singleton cases, the inheritance item has no output result. (5) Protein prediction: six tools (SIFT, PolyPhen2, MutationTaster, PrimateAI, DANN, CADD) using different methods are used to predict whether a mutation affects the structure or function of proteins. The predicting scores are in [0, 1]. A higher score means the variant is a deleterious variant with higher possibility. This filtering function can be used to select the variants whose predicting score is higher than a given value. (6) MAF: Users can set the highest MAF value in the interested populations (7) dmVar: dmVar is a mutation-disease relationship database generated from PubMed literature using text mining techniques. Choosing the red block means only show the mutations mentioned in PubMed papers. (8) Clinvar: ClinVar is a freely accessible database of the relationships among human variations and phenotypes. Red, yellow and green blocks are mean pathogenic, unknown, benign variants in Clinvar respectively. (9) OMIM: OMIM (Online Mendelian Inheritance in Man) is a database about human genes and the related genetic phenotypes. Setting this filtering option means only the genes included in OMIM are presented on the result page.

#### Gene panels

Each disease or phenotype has a predesigned gene panel which contains important genes associated with the disease or phenotype. Through selecting the specific disease or phenotype, the analysis results will focus on the genes most likely to be involved. Gene panels can minimize data analysis considerations and further reduce manual inspection work.

## Results and discussions

### Performance validation using DDD project dataset

GTX.Digest.VCF is mainly designed for WES (whole-exome sequencing) interpretation. The computing time is related to the size of VCF files. A larger VCF file requires more time to analyze. Hundreds of cases from Deciphering Developmental Disorders (DDD) project and more than 100 cases from different organizations are tested on GTX.Digest.VCF system. The interpretation process requires about 28 min for a singleton case and 52 min for a trio (family) case on average.

We compared the ranking results of our multilayer neural network with those of Exomiser and AMELIE. 305 cases with the clinical phenotype (HPO terms) and diagnosed causal gene from dataset EGAD00001001355 of DDD project are used to validate the performance of GTX.Digest.VCF. The disease-causal genes of all the selected cases are SNP variants.

For the neural network method, we adopt leave-one-out method during training process. That is, 304 VCF files were used for training, then the trained model was applied on the leave-out test VCF file. We didn’t delete any mutations or genes in VCF files before ranking during test process. For AMELIE, the corresponding genes of all mutations in each VCF file are composed of the input gene list. The newest version of Exomiser is Exomiser 11. Exomiser filters part of genes out before ranking. So some genes have no rank results in Exomiser.

**Fig. 5:**
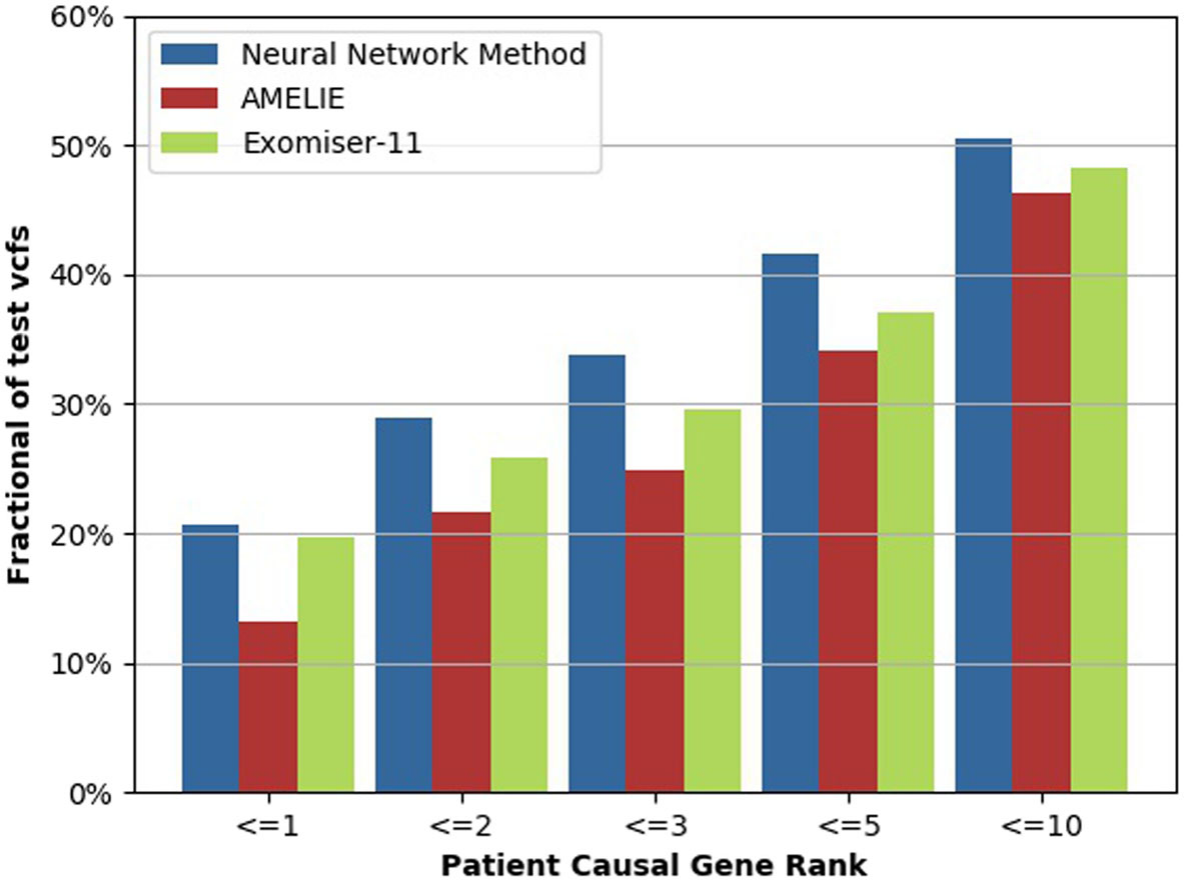
Experimental results

Figure 5 shows the results of our experiment. Ranking the causal gene is in the top 5 genes will release the burden of users for disease diagnosis. In our experiments, each VCF file contains a median of 1233 genes. Our neural network method ranks the causal gene as the very first gene to read on in 63 out of 305 cases (20.6%), and in the top 5 genes in 127 out of 305 cases (41.6%). and AMELIE and Exomiser 11 ranked the causal gene at the top in only 40 (13.1%) and 60 (19.7%) out of 305 cases, respectively. Similarly, AMELIE and Exomiser 11 ranked the causal gene in the top 5 genes in only 104 (34.1%) and 113 (37.0%) out of 305 cases, respectively.

Additionally, GTX.Digest.VCF ranks the causal gene in the top 20 genes in 190 out of 305 cases (62.3%), and in the top 50 genes in 235 out of 305 cases (77.0%). The results prove that GTX.Digest.VCF has the best accuracy of pathogenic prediction among three algorithms within this dataset.

### Case study

Users can access GTX.Digest.VCF through http://vcf.gtxlab.com for free. You can register a new account, or just log in a public account without registration. There is a demon VCF information card for a starter to quickly grasp the functions of the interpretation system. If you want to test your own cases, just click “Add a VCF file”. The system will generate a VCF information card for this case when the VCF file is uploaded correctly. You can operate the information card to input some parameters, run the case, and see the detailed interpretation results. Figure 3 is the result webpage of a typical example. The patient is a newborn with the following phenotypes:

**Table:** 

1: HP:0000465: Webbed neck 2: HP:0001520: Large for gestational age 3: HP:0001744: Splenomegaly 4: HP:0003623: Neonatal onset 5: HP:0005580: Duplication of renal pelvis 6: HP:0008752: Laryngeal cartilage malformation 7: HP:0011703: Sinus tachycardia 8: HP:0200128: Biventricular hypertrophy	

The newborn is 24 days old, and his three years old sister is healthy. The doctor diagnosed that RAF1 is the causal gene. GTX.Digest.VCF ranked RAF1 as the most pathogenic gene.

## Conclusions

GTX.Digest.VCF is a free NGS data interpretation system. Besides integrating annotation tool ANNOVAR and free databases, Clinvar and OMIM, GTX.Digest.VCF puts forward a multilayer ranking method to prioritize genes and mutations, a mined knowledge base to provide literature evidence for the relationship between mutations and diseases, and enriched filtering functions to focus on the interesting results easily and quickly. GTX.Digest.VCF shows interpretation results in many different aspects, which can reduce a lot of manual works for NGS data interpretation.

Several improvements will be integrated into the next edition of GTX.Digest.VCF in the future:

(1) FASTQ will be added as a format of genotype input and the whole running time of FASTQ-to-VCF procedure will be reduced to less than 15/30 min (WES/WGS) utilizing engineered rewrite of BWA-GATK pipeline, and FPGA hardware acceleration.

(2) Natural language processing will be used on patients’ medical records to automatically extract HPO terms as phenotype input of GTX.Digest.VCF.

(3) The identification of Copy Number Variation and Structural Variation will be added.

(4) A new phenotype-based gene ranking method is under design, which uses data mining techniques to prioritize genes and mutations. The next edition of GTX.Digest.VCF will integrate this new ranking method.

(5) Tumor-Normal pair analysis will be added as a new “Analytic type”.

### Availability and requirements

Software name: GTX.Digest.VCF.

Software access: web portal.

Software home page: http://vcf.gtxlab.com

Operating system(s): platform independent.

Other requirements: web browser, such as Google Chrome, Internet Explorer or Firefox.

## Data Availability

Supplementary material is available at http://vcf.gtxlab.com online.

## Abbreviations

AWS: Amazon web services
DDD: Deciphering developmental disorders
DTM: Distributed text mining
EFS: Elastic file system
HPO: Human phenotype ontology
NER: Named entity recognition
OMIM: Online mendelian inheritance in man
PMC: PubMed central
RE: Relation extraction
VCF: Variant call format
WES: Whole-exome sequencing

## References

[1] Li H (2011). A statistical framework for SNP calling, mutation discovery, association mapping and population genetical parameter estimation from sequencing data. Bioinformatics.

[2] Plüss M, Kopps AM, Keller I, Meienberg J, Caspar SM, Dubacher N (2017). Need for speed in accurate whole-genome data analysis: GENALICE MAP challenges BWA/GATK more than PEMapper/PECaller and Isaac. Proc. Natl. Acad. Sci. U.S.A. Nat Acad Sci.

[3] Wang K, Li M, Hakonarson H (2010). ANNOVAR: functional annotation of genetic variants from high-throughput sequencing data. Nucleic Acids Res Oxford Univ Press.

[4] Cingolani P, Platts A, Wang LL, Coon M, Nguyen T, Wang L (2012). A program for annotating and predicting the effects of single nucleotide polymorphisms, SnpEff: SNPs in the genome of *Drosophila melanogaster* strain w1118; iso-2; iso-3. Fly (Austin). Taylor & Francis.

[5] McLaren W, Gil L, Hunt SE, Riat HS, Ritchie GRS, Thormann A (2016). The Ensembl Variant Effect Predictor. Genome Biol. BioMed Central.

[6] Habegger L, Balasubramanian S, Chen DZ, Khurana E, Sboner A, Harmanci A (2012). VAT: a computational framework to functionally annotate variants in personal genomes within a cloud-computing environment. Bioinformatics.

[7] Kumar P, Henikoff S, Ng PC (2009). Predicting the effects of coding non-synonymous variants on protein function using the SIFT algorithm. Nat Protoc. Nature Publishing Group.

[8] Adzhubei IA, Schmidt S, Peshkin L, Ramensky VE, Gerasimova A, Bork P (2010). A method and server for predicting damaging missense mutations. Nat Methods Nature Publishing Group.

[9] Rentzsch P, Witten D, Cooper GM, Shendure J, Kircher M (2019). CADD: predicting the deleteriousness of variants throughout the human genome. Nucleic Acids Res.

[10] Quang D, Chen Y, Xie X (2015). DANN: a deep learning approach for annotating the pathogenicity of genetic variants. Bioinformatics.

[11] Clarke L, Zheng-Bradley X, Smith R, Kulesha E, Xiao C, Toneva I (2012). The 1000 genomes project: data management and community access. Nat. Methods. Nat Publishing Group.

[12] Koepfli K-P, Paten B (2015). Genome 10K Community of Scientists, O'Brien SJ. The genome 10K project: a way forward. Annu rev Anim Biosci. Annu Rev.

[13] Karczewski KJ, Weisburd B, Thomas B, Solomonson M, Ruderfer DM, Kavanagh D (2017). The ExAC browser: displaying reference data information from over 60 000 exomes. Nucleic Acids Res.

[14] Auer PL, Johnsen JM, Johnson AD, Logsdon BA, Lange LA, Nalls MA (2012). Imputation of exome sequence variants into population- based samples and blood-cell-trait-associated loci in African Americans: NHLBI GO exome sequencing project. Am J Hum Genet.

[15] Köhler S, Doelken SC, Mungall CJ, Bauer S, Firth HV, Bailleul-Forestier I (2014). The human phenotype ontology project: linking molecular biology and disease through phenotype data. Nucleic Acids Res.

[16] Smedley D, Jacobsen JOB, Jäger M, Köhler S, Holtgrewe M, Schubach M (2015). Next-generation diagnostics and disease-gene discovery with the exomiser. Nat Protoc Nat Publishing Group.

[17] Johannes Birgmeier A, Haeussler M, Deisseroth CA, Jagadeesh KA, Ratner AJ, Guturu H, et al. AMELIE accelerates Mendelian patient diagnosis directly from the primary literature. bioRxiv. 2017:1–23.

[18] Köhler S, Schulz MH, Krawitz P, Bauer S, Dölken S, Ott CE (2009). Clinical diagnostics in human genetics with semantic similarity searches in ontologies. Am J Hum Genet.

[19] Singleton MV, Guthery SL, Voelkerding KV, Chen K, Kennedy B, Margraf RL (2014). Phevor combines multiple biomedical ontologies for accurate identification of disease-causing alleles in single individuals and small nuclear families. Am J Hum Genet.

[20] Yang H, Robinson PN, Wang K (2015). Phenolyzer: phenotype-based prioritization of candidate genes for human diseases. Nat Methods.

[21] Smedley D, Robinson PN (2015). Phenotype-driven strategies for exome prioritization of human Mendelian disease genes. Genome Med.

[22] Robinson PN, Kohler S, Oellrich A, Wang K, Mungall CJ, Lewis SE (2014). Improved exome prioritization of disease genes through cross-species phenotype comparison. Genome Res.

[23] Haendel MA, Vasilevsky N, Brush M, Hochheiser HS, Jacobsen J, Oellrich A (2015). Disease insights through cross-species phenotype comparisons. Mamm Genome.

[24] Amberger Joanna S., Bocchini Carol A., Schiettecatte François, Scott Alan F., Hamosh Ada (2014). OMIM.org: Online Mendelian Inheritance in Man (OMIM®), an online catalog of human genes and genetic disorders. Nucleic Acids Research.

[25] Pavan S, Rommel K, Marquina MEM, Höhn S, Lanneau V, Rath A (2017). Clinical practice guidelines for rare diseases: the Orphanet database. PLoS One.

[26] Allot A, Peng Y, Wei C-H, Lee K, Phan L, Lu Z (2018). LitVar: a semantic search engine for linking genomic variant data in PubMed and PMC. Nucleic Acids Res.

[27] Singhal A, Simmons M, Lu Z. Text Mining Genotype-Phenotype Relationships from Biomedical Literature for Database Curation and Precision Medicine. Rzhetsky A, editor. PLoS Comput. Biol. Public Libr Sci. 2016;12:e1005017. 10.1371/journal.pcbi.1005017 PMC5130168 27902695

[28] Zhang Y, Shen F, Mojarad MR, Li D, Liu S, Tao C, et al. Systematic identification of latent disease-gene associations from PubMed articles. Bajic VB, editor. PLoS ONE. Public Libr Sci. 2018;13:e0191568. 10.1371/journal.pone.0191568 PMC5786305 29373609

[29] Landrum MJ, Lee JM, Benson M, Brown G, Chao C, Chitipiralla S (2016). ClinVar: public archive of interpretations of clinically relevant variants. Nucleic Acids Res.

[30] Margulies EH, Blanchette M, Thomas J, Touchman J, Blakesley B (2003). Identification and characterization of multi-species conserved sequences. Genome Res.

[31] Fang C, Noguchi T, Yamana H (2014). Analysis of evolutionary conservation patterns and their influence on identifying protein functional sites. J Bioinforma Comput Biol.

[32] Davydov EV, Goode DL, Sirota M, Cooper GM, Sidow A (2010). Identifying a high fraction of the human genome to be under selective constraint using GERP++. PLoS Comput Biol.

[33] Leaman R, Islamaj Dogan R, Lu Z (2013). DNorm: disease name normalization with pairwise learning to rank. Bioinformatics Oxford University Press.

[34] Wei C-H, Phan L, Feltz J, Maiti R, Hefferon T, Lu Z (2017). tmVar 2.0: integrating genomic variant information from literature with dbSNP and ClinVar for precision medicine. Bioinformatics.

[35] Song M, Kim WC, Lee D, Heo GE, Kang KY (2015). PKDE4J: entity and relation extraction for public knowledge discovery. J Biomed Inform.

